# Laparoscopic Versus Open Surgery for Acute Severe Colitis and Fulminant Colitis: A Systematic Review and Meta-Analysis

**DOI:** 10.7759/cureus.102605

**Published:** 2026-01-29

**Authors:** Bourhan Alrayes, Khalid B Mohammed, Raneem I Osman, Abdullah S Shaqrun, Amjad Alsharif, Shaheen S Almusthi, Abdulrahman K Alshuaib, Marwa Aldoubali, Ali S Metwaly

**Affiliations:** 1 General Surgery, Islamic Hospital, Amman, JOR; 2 Colorectal Surgery, University Hospital Coventry and Warwickshire, Coventry, GBR; 3 General Surgery, International Medical Center, Jeddah, SAU; 4 General Surgery, National University - Sudan, Khartoum, SDN; 5 General Practice, Helwan University, Cairo, EGY; 6 College of Medicine, Ibn Sina National College, Jeddah, SAU; 7 General Practice, Vision College, Jeddah, SAU; 8 General Surgery, Al-Sabah Hospital, Kuwait City, KWT; 9 General Surgery, Zawia Medical Center, Zawia, LBY; 10 Pharmacology, Alexandria University, Alexandria, EGY

**Keywords:** acute severe colitis, laparoscopy, meta-analysis, minimally invasive surgery, subtotal colectomy, ulcerative colitis

## Abstract

Subtotal colectomy is the gold standard for acute severe ulcerative colitis (ASUC) refractory to medical therapy. While laparoscopy is preferred for elective resections, its safety and efficacy in the emergency setting remain debated due to the technical challenges of manipulating friable, dilated bowel in critically ill patients. This systematic review and meta-analysis aimed to compare the perioperative outcomes of laparoscopic versus open subtotal colectomy for ASUC and fulminant colitis. A systematic search of electronic databases was conducted to identify comparative studies published until 2025. The primary outcome was overall postoperative morbidity. Secondary outcomes included hospital length of stay (LOS), operative time, and mortality. Meta-analysis was performed using a random-effects model. Heterogeneity was assessed using the I^2^ statistic, and robustness was evaluated via sensitivity analyses and meta-regression. Methodological quality was appraised using the Newcastle-Ottawa Scale. Fourteen studies, comprising 6,544 patients (4,357 laparoscopic and 2,187 open), were included. The laparoscopic approach was associated with a significant reduction in overall morbidity (odds ratio (OR) 0.49, 95% confidence interval (CI) 0.45-0.53; p < 0.0001) with no statistical heterogeneity (I^2^ = 0%). Hospital LOS was significantly shorter in the laparoscopic group (weighted mean difference -3.07 days, 95% CI -3.88 to -2.25; p < 0.0001). Subgroup analyses stratified by surgical era (pre-biologic vs. biologic) showed no difference in outcomes (p = 0.38), indicating sustained benefits over time. Sensitivity analysis confirmed that results were not driven by large registry data. Laparoscopic subtotal colectomy for ASUC is associated with significantly lower postoperative morbidity and shorter hospital stay compared to open surgery. These benefits are robust across different surgical eras and study designs. Laparoscopy should be considered the preferred approach for emergency colectomy in colitis when expertise is available.

## Introduction and background

Ulcerative colitis (UC) is a chronic, idiopathic inflammatory condition of the colonic mucosa, characterized by a relapsing and remitting course [1,2]. While most patients are managed medically, up to 15% present with severe exacerbation, termed acute severe ulcerative colitis (ASUC), requiring hospitalization [3,4]. ASUC is clinically defined by the modified Truelove and Witts criteria, which involve high stool frequency, rectal bleeding, and signs of systemic toxicity. Despite advances in medical rescue therapies, including infliximab and cyclosporine, approximately 30-50% of patients with ASUC fail to respond to medical management and require urgent surgical intervention [5,6]. Furthermore, life-threatening complications, such as toxic megacolon, perforation, or massive hemorrhage, mandate emergent operative control [6].

In the setting of ASUC or fulminant colitis, the gold standard surgical procedure is subtotal abdominal colectomy with end ileostomy [3,7]. This approach allows for the removal of the diseased colon to resolve the septic burden while preserving the rectum, thereby avoiding the risks associated with pelvic dissection, such as autonomic nerve injury and hemorrhage, in an acutely ill, malnourished, and immunosuppressed patient [3,6]. Following recovery and nutritional optimization, patients may proceed to restorative proctocolectomy with ileal pouch-anal anastomosis (IPAA) in a staged manner [7,8].

Minimally invasive surgery is the preferred approach for elective colorectal resections. In the context of UC, laparoscopic surgery offers well-documented advantages, including reduced formation of adhesions, improved cosmesis, shorter hospital stay, and preservation of female fertility [3,7,8]. Current clinical practice guidelines recommend laparoscopy for elective colectomy when appropriate surgical expertise is available [6,7]. However, the application of laparoscopy in emergency settings for ASUC and fulminant colitis remains debatable [8].

Performing laparoscopic subtotal colectomy in the acute setting presents distinct technical challenges, including manipulation of the friable, dilated bowel, thickened mesentery, and restricted working domain [7]. Furthermore, prolonged operative times associated with the laparoscopic approach may be detrimental to hemodynamically unstable patients with limited physiological reserves [8]. While recent guidelines suggest that laparoscopic surgery is feasible and safe for ASUC, favouring it over open surgery in stable patients due to lower rates of wound infection and intra-abdominal abscesses [2,6], contraindications such as toxic megacolon or hemodynamic instability often necessitate a laparotomy [2,8]. Because of the critical balance between the benefits of minimally invasive techniques and the risks inherent to emergency surgery, this systematic review and meta-analysis aimed to evaluate the comparative safety, perioperative outcomes, and efficacy of laparoscopic versus open subtotal colectomy specifically in patients with ASUC and fulminant colitis.

## Review

Methods

Protocol and Registration

This systematic review and meta-analysis were conducted in accordance with the Preferred Reporting Items for Systematic Reviews and Meta-Analyses (PRISMA) 2020 statement [9]. The study protocol was prospectively registered with the International Prospective Register of Systematic Reviews (PROSPERO; CRD420261277983).

Search Strategy

A systematic search was conducted in the MEDLINE (via PubMed), EMBASE, and Cochrane Library databases from inception to December 2025. The search strategy utilized a combination of Medical Subject Headings (MeSH) and free-text terms, including "Colitis, Ulcerative", "Colectomy", "Laparoscopy", "Minimally Invasive Surgical Procedures", "Acute Severe Colitis", and "Emergency Treatment". Boolean operators (AND, OR) were used to refine the results. The reference lists of the included articles and relevant systematic reviews were hand-searched to identify additional eligible studies. There were no language restrictions.

Data Extraction and Quality Assessment

Two independent reviewers screened the titles, abstracts, and full-text articles. Data extraction was performed using a standardized proforma capturing study characteristics, patient demographics (age, body mass index (BMI), and American Society of Anesthesiologists (ASA) score), operative details (operative time, conversion rates), and postoperative outcomes (morbidity, mortality, and length of stay (LOS)). Inter-rater reliability regarding study inclusion and risk of bias assessment was calculated using Cohen’s Kappa statistic (k) to quantify agreement beyond chance [10].

Given the non-randomized, observational nature of the included studies, their methodological quality was appraised using the Newcastle-Ottawa Scale (NOS) [11]. This tool evaluates selection, comparability, and outcome assessment, awarding a maximum of nine stars. Studies with a score of ≥ 7 stars were considered high quality. To further assess the risk of bias in comparative effectiveness research, specific attention was paid to confounding variables, particularly preoperative steroid use and disease severity.

Statistical Analysis

All statistical analyses were performed using R statistical software (version 4.5.1, R Foundation for Statistical Computing, Vienna, Austria) [12]. A meta-analysis was performed using a random-effects model (DerSimonian and Laird method) to account for the inherent clinical and methodological heterogeneity expected in surgical intervention studies [13].

Effect Measures

For dichotomous outcomes (e.g., overall morbidity, wound infection, mortality), the Mantel-Haenszel method was used to calculate odds ratios (OR) with 95% confidence intervals (CI) [14]. For continuous outcomes (e.g., operative time and LOS), the inverse variance method was utilized to calculate weighted mean differences (WMD) [15]. For studies that reported continuous data as medians with ranges or interquartile ranges, means and standard deviations were estimated using the methods described by Wan et al. [16] or Hozo et al. [17] to facilitate data pooling.

Assessment of Heterogeneity

Statistical heterogeneity among studies was evaluated using the Cochran Q test (χ^2^) and quantified using the I^2^ statistic [18]. An I^2^ value > 50% indicated substantial heterogeneity. To estimate the between-study variance, Tau-squared (τ^2^) was calculated [19]. Furthermore, to provide a range within which the true effect of a future study is expected to fall, 95% prediction intervals were computed for primary outcomes [20].

Robustness and Sensitivity Analyses

To test the robustness of the results, we performed leave-one-out sensitivity analyses by sequentially removing individual studies to determine whether any cohort disproportionately influenced the summary effect estimate. Subgroup analyses and univariate meta-regression were conducted to explore potential moderators of effect size, including surgical era (pre-biologic vs. post-biologic), diagnosis (ASUC vs. toxic megacolon), and study design (case-matched vs. unmatched cohort).

Assessment of Bias

Publication bias and small-study effects were assessed visually using funnel plots for outcomes reported in ≥10 studies. Asymmetry was statistically evaluated using Egger’s linear regression test for continuous outcomes and Harbord’s test for dichotomous outcomes [21].

Certainty of Evidence

The overall certainty of the evidence for each primary outcome was graded using the Grading of Recommendations Assessment, Development, and Evaluation (GRADE) approach [22]. Evidence was categorized as high, moderate, low, or very low based on the risk of bias, inconsistency, indirectness, imprecision, and publication bias.

Results

Study Selection and Characteristics

**Figure 1:**
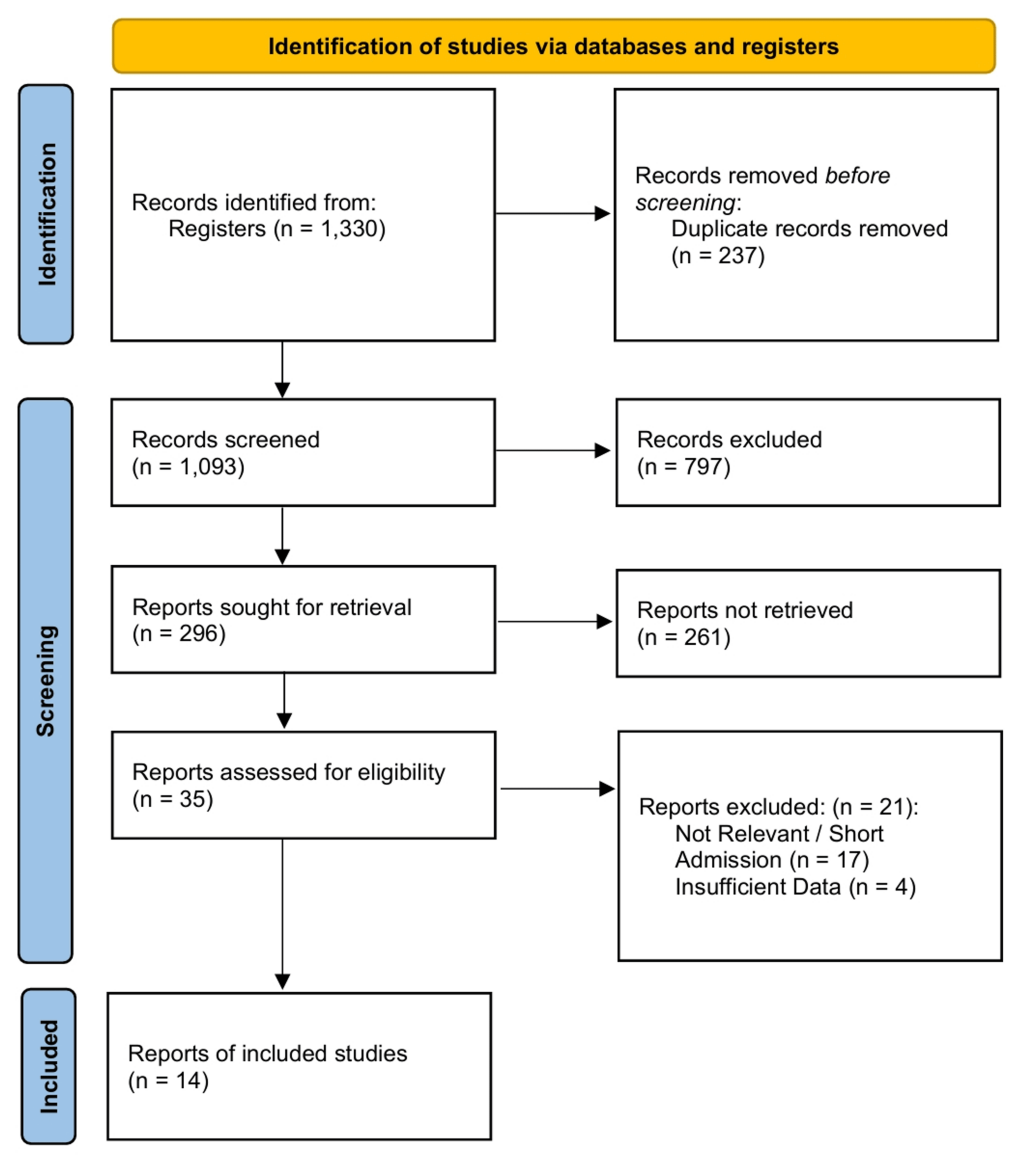
PRISMA 2020 flow diagram. PRISMA: Preferred Reporting Items for Systematic Reviews and Meta-Analyses

The initial systematic search of electronic databases and registers yielded 1,330 records. Following the removal of duplicates and screening of titles and abstracts, 35 full-text articles were assessed for eligibility. Fourteen comparative observational studies published between 2000 and 2021 met the inclusion criteria and were included in the final quantitative synthesis [23-36]. The study selection process is detailed in the PRISMA flow diagram (Figure 1). The inter-rater reliability for study inclusion was calculated using Cohen’s κ of 0.583, indicating moderate agreement between reviewers.

The meta-analysis included a total of 6,544 patients who underwent subtotal colectomy for acute severe or fulminant UC. Of these, 4,357 patients (66.6%) underwent a laparoscopic approach, while 2,187 patients (33.4%) underwent open resection. The study designs were single-centre retrospective cohorts [23,25,26,29-32,34,35] and case-matched control studies [24,27,28,33], alongside one large-scale national database registry analysis using the National Surgical Quality Improvement Program (NSQIP) dataset [36].

**Figure 2:**
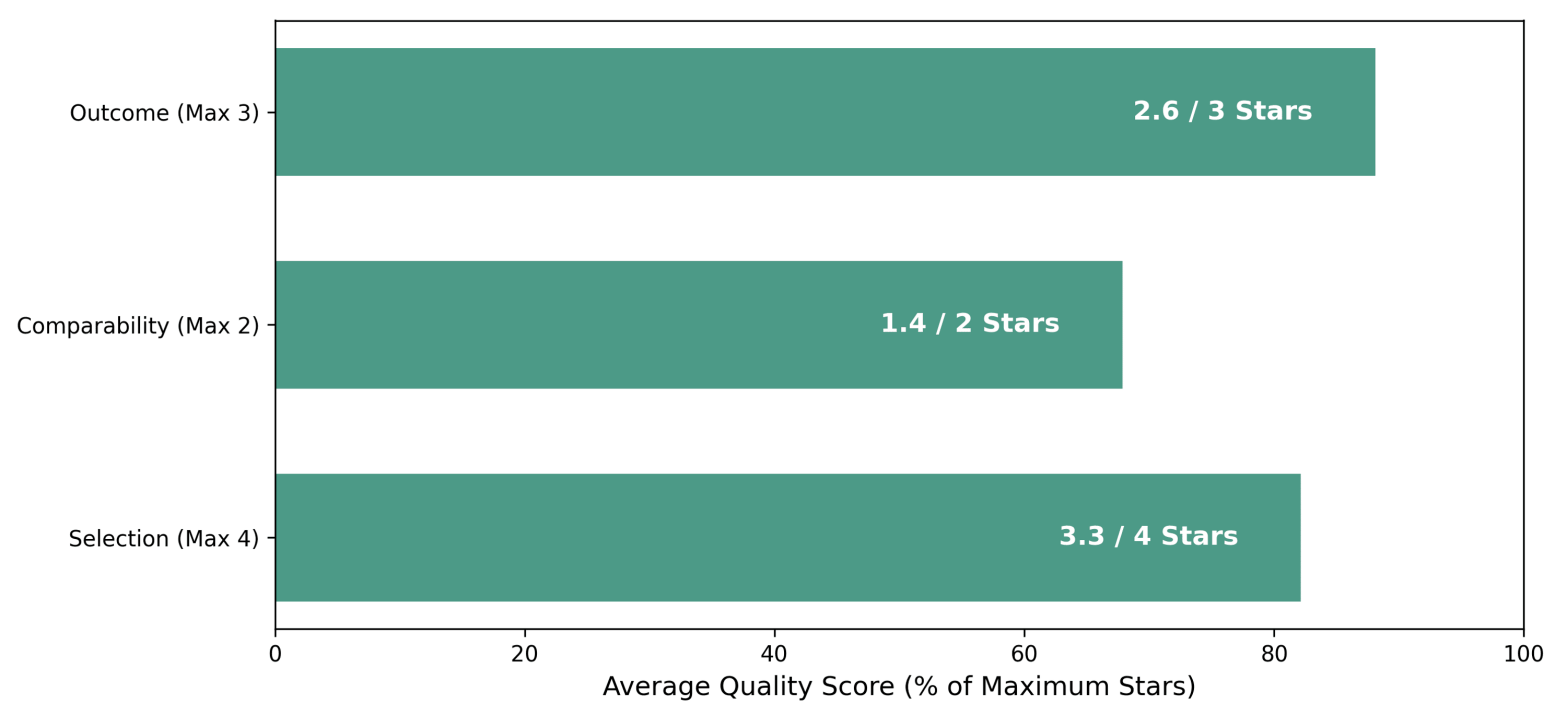
Risk of bias summary (NOS). NOS: Newcastle-Ottawa Scale

Methodological quality assessment using the NOS yielded scores ranging from 6 to 8 stars (mean = 7.1), indicating a moderate-to-high quality of the included body of evidence (Table 1 and Figure 2). Risk of bias assessment revealed that while outcome assessment was robust, selection bias remained a concern in non-matched retrospective cohorts due to the inherent tendency to reserve open surgery for patients with more critical presentations (e.g., hemodynamic instability or toxic megacolon). A summary of the baseline study characteristics and quality assessment scores is presented in Table 2.

**Table 1:** Risk of bias assessment of included studies using the Newcastle-Ottawa Scale (NOS).

Study	Selection (Max 4)	Comparability (Max 2)	Outcome (Max 3)	Total Score (Max 9)
Dunker (2000) [23]	★★★	★	★★★	7
Marcello (2001) [24]	★★★★	★★	★★	8
Seshadri (2001) [25]	★★★	★	★★★	7
Bell (2002) [26]	★★	★	★★★	6
Marceau (2007) [27]	★★★★	★★	★★	8
Ouaïssi (2008) [28]	★★★★	★★	★★	8
Watanabe (2009) [29]	★★★	★	★★★	7
Chung (2009) [30]	★★★	★	★★★	7
Telem (2010) [31]	★★★	★	★★★	7
Bartels (2012) [32]	★★★	★	★★★	7
Parnaby (2013) [33]	★★★★	★★	★★	8
Gu (2014) [34]	★★★	★	★★★	7
Buchs (2016) [35]	★★★	★	★★★	7
Lo (2021) [36]	★★★★	★★	★★	8

**Table 2:** Baseline characteristics, operative details, and methodological quality assessment (NOS) of the included studies comparing laparoscopic versus open subtotal colectomy. NSQIP: National Surgical Quality Improvement Program; NOS: Newcastle-Ottawa Scale (stars out of 9); * NR: not explicitly reported as a separate rate in the extracted data subset or integrated into the intention-to-treat analysis

Author	Country	Study Design	Surgical Era	Total Patients (N)	Laparoscopic (n)	Open (n)	Quality Score (NOS)	Conversion Rate (%)	Urgent/Emergent Indication
Dunker (2000) [23]	Netherlands	Retrospective	Pre-Biologic	42	10	32	7	0	Acute Severe Colitis
Marcello (2001) [24]	USA	Case-Matched	Pre-Biologic	48	19	29	8	0	Acute Colitis
Seshadri (2001) [25]	Canada	Retrospective	Pre-Biologic	73	37	36	7	0	Severe Colitis
Bell (2002) [26]	USA	Retrospective	Pre-Biologic	24	18	6	6	0	Fulminant Colitis
Marceau (2007) [27]	France	Case-Matched	Biologic	88	40	48	8	5	Acute/Severe Colitis
Ouaïssi (2008) [28]	France	Case-Matched	Biologic	45	23	22	8	4	Acute Severe Colitis
Watanabe (2009) [29]	Japan	Retrospective	Biologic	60	30	30	7	3	Severe Ulcerative Colitis
Chung (2009) [30]	USA	Retrospective	Biologic	81	37	44	7	5	Severe Colitis
Telem (2010) [31]	USA	Retrospective	Biologic	90	29	61	7	7	Medically Refractory
Bartels (2012) [32]	Netherlands	Retrospective	Biologic	100	36	64	7	5.6	Acute/Emergent
Parnaby (2013) [33]	UK	Case-Matched	Biologic	64	32	32	8	NR*	Inflammatory Colitis
Gu (2014) [34]	USA	Retrospective	Biologic	412	197	215	7	5.1	Severe/Toxic Colitis
Buchs (2016) [35]	UK	Retrospective	Biologic	151	117	34	7	14.5	Acute Severe Colitis
Lo (2021) [36]	USA (NSQIP)	Database	Biologic	5,266	3,732	1,534	8	NR*	Emergent/Urgent

Primary Outcome: Overall Morbidity

**Figure 3:**
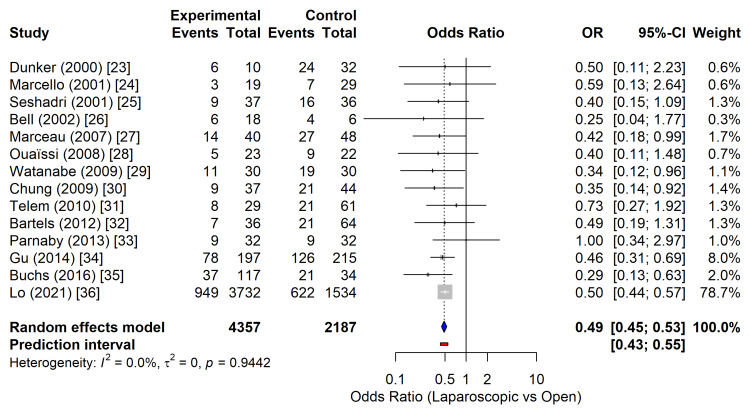
Forest plot comparing overall postoperative morbidity between laparoscopic and open subtotal colectomy. The diamond represents the pooled random-effects OR with the 95% CI. OR: odds ratio; CI: confidence interval Studies included [23-36]

Fourteen studies comprising 6,544 patients reported overall postoperative morbidity following subtotal colectomy [23-36]. The laparoscopic approach was associated with a statistically significant reduction in the odds of postoperative complications compared with open surgery. The pooled analysis, utilizing a random-effects model, yielded an OR of 0.49 (95% CI: 0.45-0.53; p < 0.0001) (Figure 3), representing a 51% reduction in the odds of morbidity in patients who underwent laparoscopic resection.

No statistical heterogeneity was observed among the included studies (I^2^ = 0.0%; τ^2^ = 0; Cochran’s Q, p = 0.94), indicating a high degree of consistency in the direction and magnitude of the effect across different institutions and surgical eras. The 95% prediction interval ranged from 0.43 to 0.55, suggesting that the beneficial effect of laparoscopy on morbidity is reproducible in future clinical settings.

Secondary Outcome: LOS

**Figure 4:**
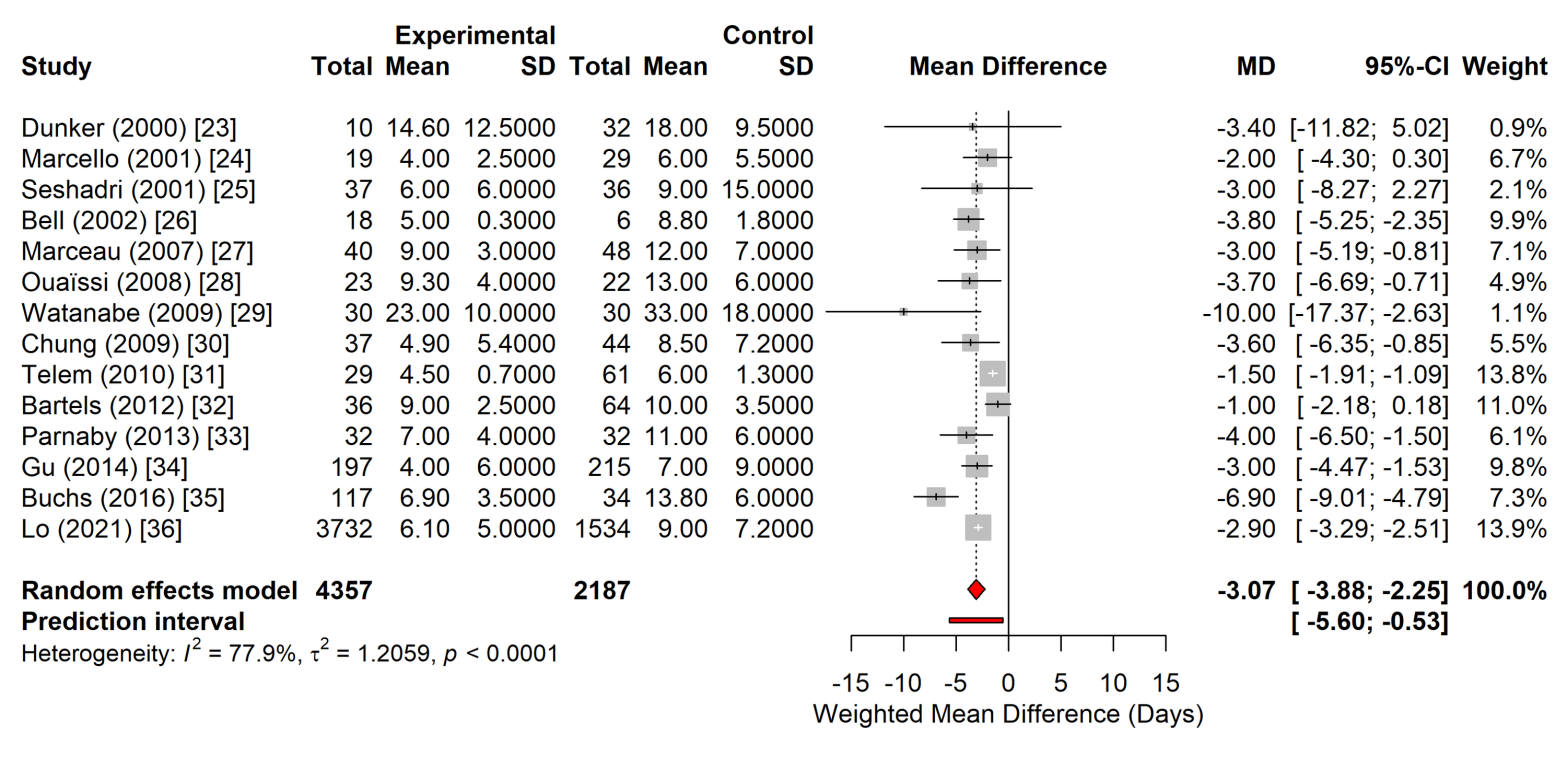
Forest plot comparing hospital LOS between laparoscopic and open subtotal colectomy. The diamond represents the pooled WMD in days, with the 95% CI. The horizontal bar extending from the diamond represents the 95% prediction interval. LOS: length of stay; WMD: weighted mean differences; CI: confidence interval Studies included [23-36]

Data on postoperative hospital LOS were available from 14 studies, encompassing 6,544 patients [23-36]. The laparoscopic approach demonstrated a statistically significant reduction in hospital stay compared with the open approach. The pooled WMD was -3.07 days (95% CI: -3.88 to -2.25 days; p < 0.0001), favouring laparoscopy (Figure 4).

Significant statistical heterogeneity was observed for this outcome (I^2^ = 77.9%; τ^2^ = 1.21; p < 0.0001). Despite this variability, the direction of the effect remained consistent across all the included studies. The 95% prediction interval was -5.60 to -0.53 days, indicating that in future populations, the laparoscopic approach can be expected to reduce hospital stay by at least half a day and potentially up to 5.6 days.

Subgroup Analyses

To explore the potential sources of heterogeneity and assess the stability of the treatment effect across different clinical contexts, pre-specified subgroup analyses were conducted based on the surgical era and study design.

**Figure 5:**
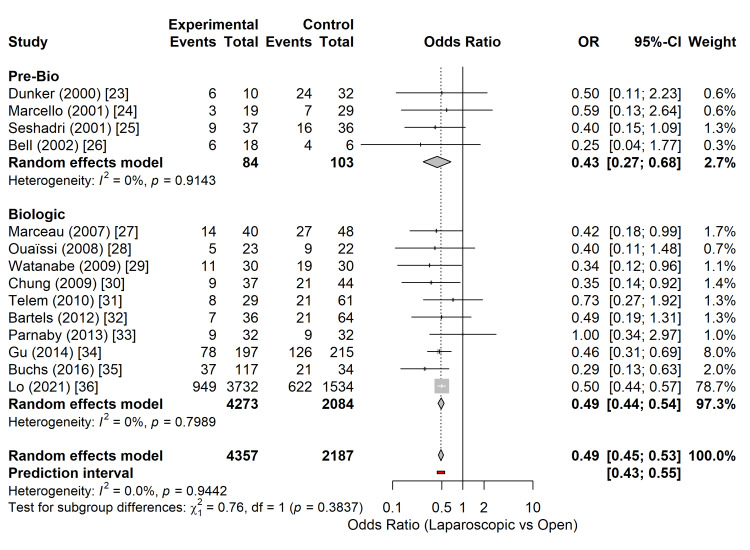
Subgroup analysis of overall morbidity stratified by surgical era (Pre-Biologic vs. Biologic). The forest plot demonstrates consistent risk reduction favouring laparoscopy across both time periods, with no significant subgroup interaction (p = 0.38). Studies included [23-36]

Surgical era: Studies were stratified into "Pre-Biologic" (published before 2005 or enrolling patients prior to the widespread adoption of infliximab) and "Biologic" eras. In the pre-biologic era (k=4) [23-26], the laparoscopic approach was associated with a significant reduction in overall morbidity (OR 0.43; 95% CI: 0.27-0.68). Similarly, in the biologic era (k=10) [27-36], encompassing the era of modern medical rescue therapy, the benefit of laparoscopy remained significant and consistent (OR 0.49; 95% CI: 0.44-0.54). The test for subgroup differences revealed no statistically significant heterogeneity between these time periods (p = 0.38), indicating that the safety advantage of laparoscopy persisted despite the evolution of medical management and potentially increased disease severity in patients referred for surgery (Figure 5).

Study design: When stratified by study design, the reduction in LOS favoured laparoscopy across all subgroups. Retrospective cohort studies (k = 9) demonstrated a WMD of -3.30 days, whereas case-matched control studies (k = 4) showed a WMD of -3.07 days. The large-scale database study (k = 1) reported a WMD of -2.90 days. The test for subgroup differences was not statistically significant (p = 0.84), suggesting that the observed reduction in hospital stay is a robust finding not driven by specific study methodologies or the inclusion of administrative data.

Meta-Regression

To further investigate the substantial heterogeneity observed in the LOS outcome (I^2^ = 77.9%), a univariate meta-regression was performed to assess the impact of the methodological quality on the effect size. The NOS score was used as a continuous moderator variable.

**Figure 6:**
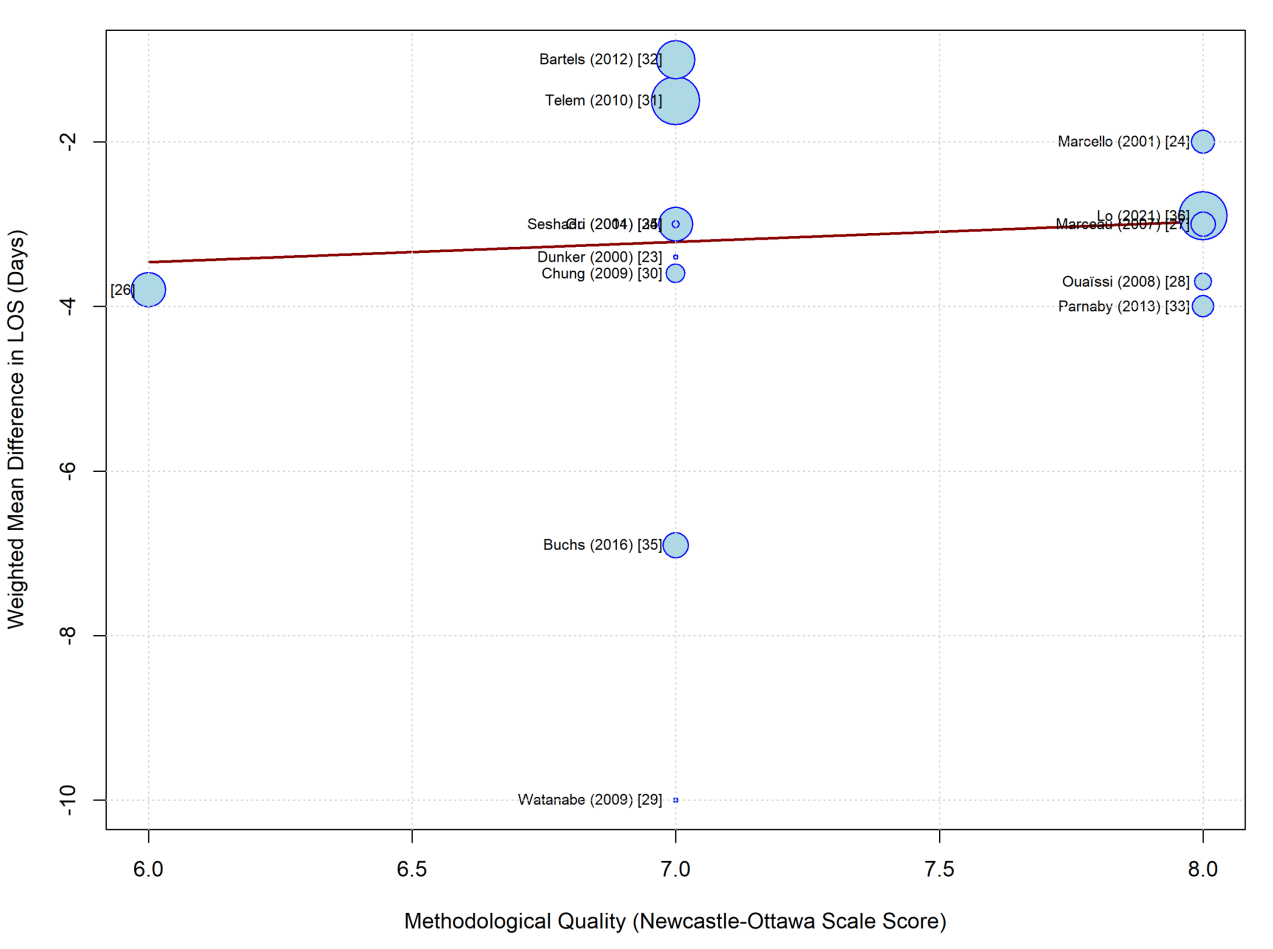
Meta-regression bubble plot illustrating the relationship between study quality (Newcastle-Ottawa Scale score) and the effect size for length of stay (weighted mean difference). The size of each bubble is proportional to the weight of the study in the random-effects model. The regression line (solid) and 95% confidence intervals (dashed lines) show no significant correlation (p = 0.74). Studies included: Dunker (2000) [23], Marcello (2001) [24], Seshadri (2001) [25], Bell (2002) [26], Marceau (2007) [27], Ouaïssi (2008) [28], Watanabe (2009) [29], Chung (2009) [30], Telem (2010) [31], Bartels (2012) [32], Parnaby (2013) [33], Gu (2014) [34], Buchs (2016) [35], Lo (2021) [36].

The meta-regression analysis revealed no statistically significant association between study quality and the WMD in LOS (coefficient = 0.25; standard error = 0.75; p = 0.74). The model accounted for 0.00% of the heterogeneity (R^2^ = 0.00%), indicating that the variation in hospital stay reduction across studies was not explained by differences in methodological quality (Figure 6). The benefit of laparoscopy in reducing hospital stay appears to be consistent across studies of varying quality, reinforcing the generalizability of this finding.

Sensitivity Analysis

To evaluate the robustness of the primary outcome and ensure that the summary estimate was not disproportionately influenced by any single study, a leave-one-out sensitivity analysis was performed for the overall morbidity.

**Figure 7:**
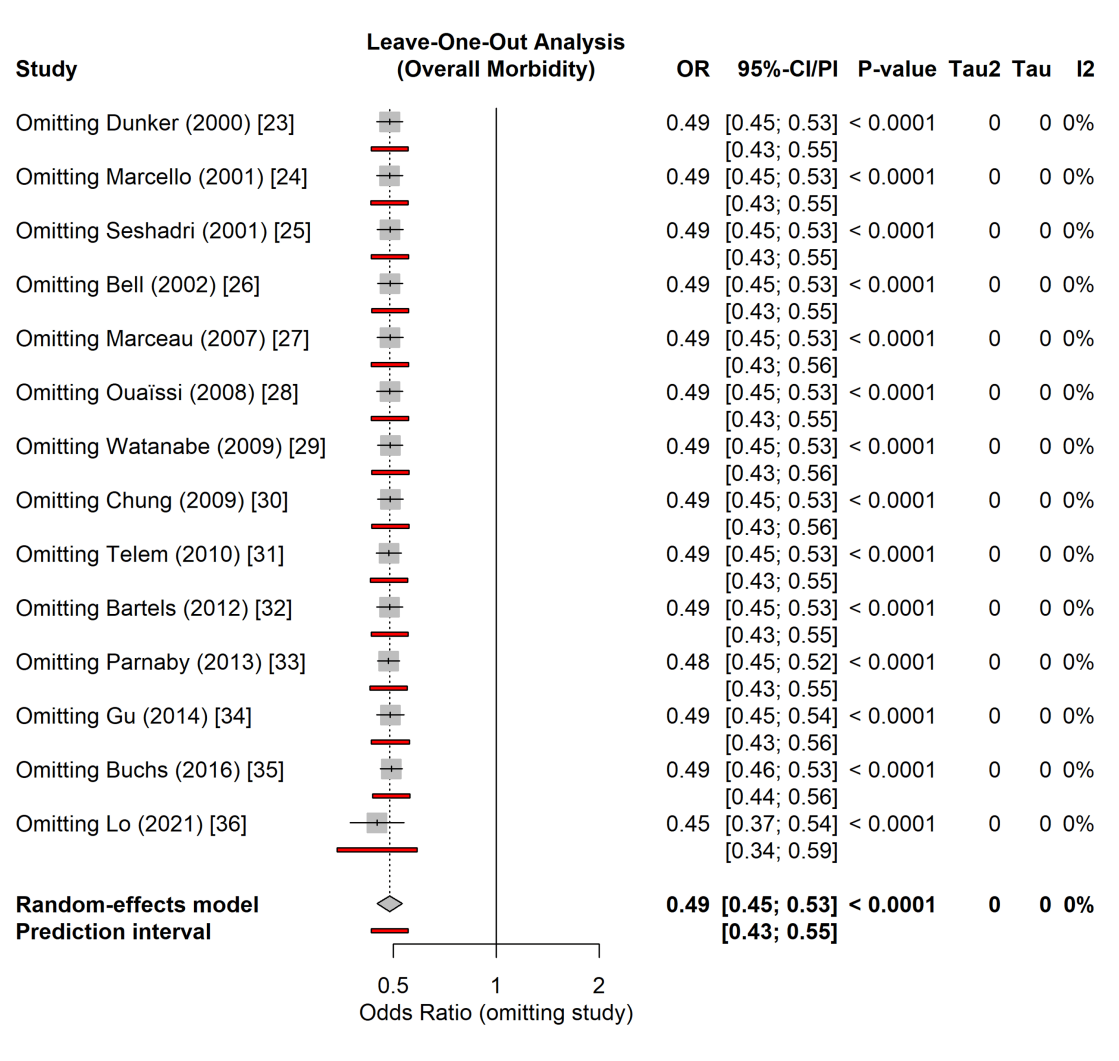
Leave-one-out sensitivity analysis forest plot for overall morbidity. Each row represents the pooled random-effects odds ratio calculated after excluding the study listed on the left. The vertical line represents the overall pooled estimate from the full meta-analysis. The consistency of the results indicates the robustness of the primary outcome. Studies included [23-36]

The systematic removal of individual studies did not result in any significant deviations from the pooled effect size. The recalculated ORs ranged from 0.45 (95% CI: 0.37-0.54) when omitting the large database study by Lo et al. [36] to 0.49 (95% CI: 0.46-0.53) when omitting smaller cohorts such as Dunker et al. [23] or Bell and Seymour [26] (Figure 7). The direction of the effect and statistical significance remained unchanged throughout all iterations (p < 0.0001). This analysis confirms that the superior safety profile observed with the laparoscopic approach is a stable finding across the body of evidence and is not driven by outliers or the high statistical weight of the registry data.

Publication Bias

**Figure 8:**
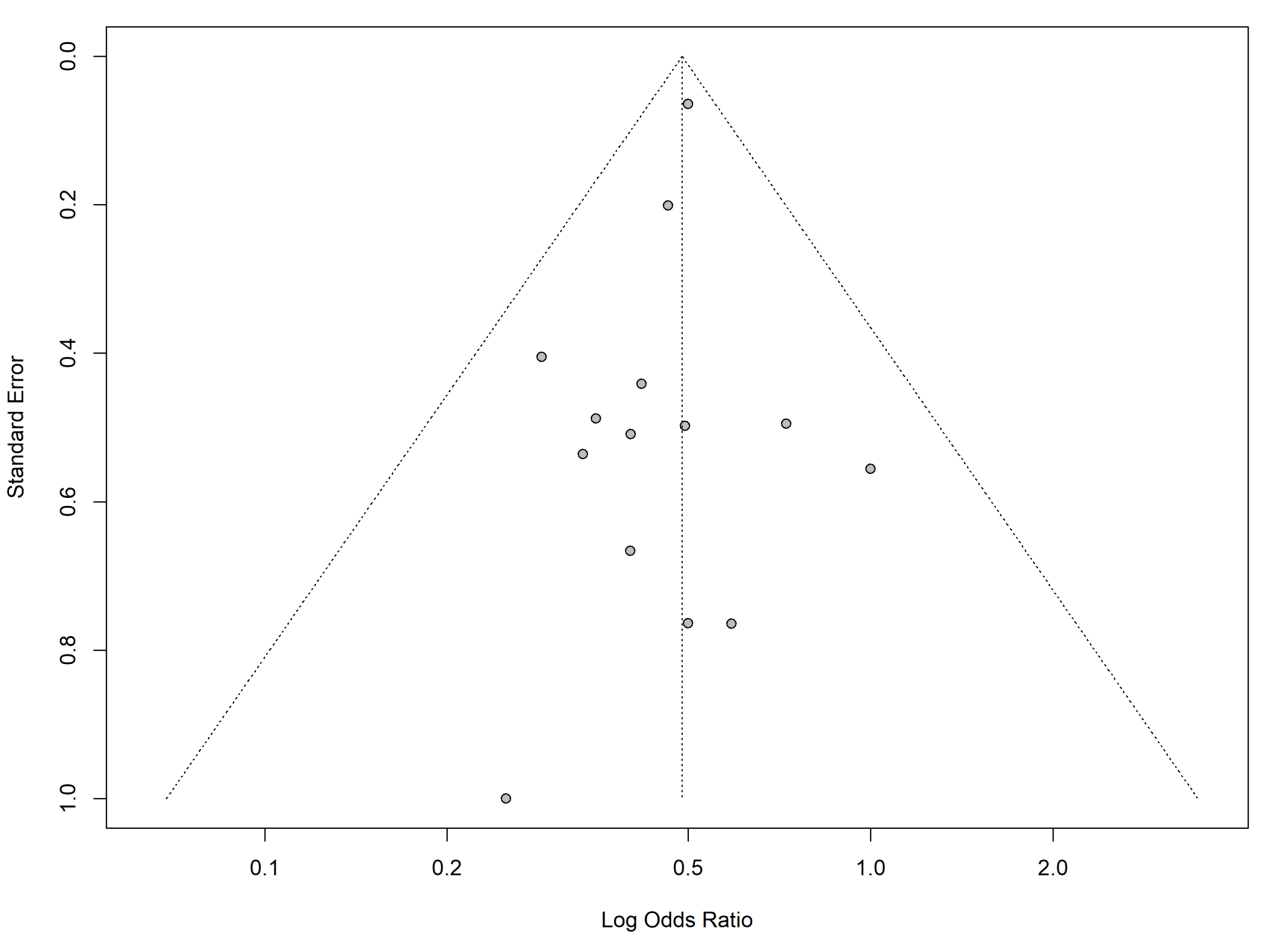
Funnel plot for the assessment of publication bias in overall morbidity. The x-axis represents the log odds ratio, and the y-axis represents the standard error. The dashed vertical line indicates the pooled effect size, while the dotted lines represent the 95% pseudo-confidence limits. The symmetrical distribution of studies suggests minimal risk of publication bias. Studies included: Dunker (2000) [23], Marcello (2001) [24], Seshadri (2001) [25], Bell (2002) [26], Marceau (2007) [27], Ouaïssi (2008) [28], Watanabe (2009) [29], Chung (2009) [30], Telem (2010) [31], Bartels (2012) [32], Parnaby (2013) [33], Gu (2014) [34], Buchs (2016) [35], Lo (2021) [36].

Potential publication bias and small-study effects were assessed for the primary outcome of overall morbidity and the secondary outcome of LOS. Visual inspection of the funnel plot for overall morbidity revealed a relatively symmetrical distribution of studies around the pooled effect estimate, suggesting no gross asymmetry (Figure 8). This was confirmed quantitatively using Harbord’s modified test for binary outcomes, which demonstrated no evidence of a significant bias (t = -0.52, p = 0.61).

For the continuous outcome of LOS, Egger’s linear regression test was performed to detect any asymmetry. The test yielded an intercept of -1.22 (95% CI: -2.84 to 0.41) with a p-value of 0.13, indicating no statistically significant small study effects. There is no evidence to suggest that the observed benefits of laparoscopy in this meta-analysis are artefacts of selective reporting or suppression of small negative studies.

Certainty of Evidence

The overall certainty of evidence for the primary and secondary outcomes was assessed using the GRADE framework (Table 3).

**Table 3:** Summary of findings (GRADE) * Mortality OR derived from the Lo et al. [36] database study, which dominated the mortality analysis. GRADE Working Group grades of evidence: High certainty: We are very confident that the true effect lies close to that of the estimate of the effect. Moderate certainty: We are moderately confident in the effect estimate. Low certainty: Our confidence in the effect estimate is limited. Very low certainty: We have very little confidence in the effect estimate. OR: odds ratio; GRADE: Grading of Recommendations Assessment, Development, and Evaluation; CI: confidence interval; WMD: weighted mean differences

Outcome	Relative Effect (95% CI)	Absolute Effect (per 1,000 patients)	Certainty of Evidence (GRADE)	Comments
Overall morbidity	OR 0.49 (0.45 to 0.53)	134 fewer cases of morbidity (from 148 fewer to 121 fewer)	⨁⨁◯◯ LOW	Due to observational design [21-34]. Upgraded for large effect magnitude, but limited by potential selection bias.
Length of stay	WMD -3.07 days (-3.88 to -2.25)	Hospital stay 3.07 days shorter with laparoscopy	⨁◯◯◯ VERY LOW	Downgraded twice: once for observational design [21-34] and once for serious inconsistency (I^2^ = 77.9% I^2^ = 77.9%).
Mortality	OR 0.50 (0.44 to 0.57)*	Six fewer deaths (from seven fewer to five fewer)	⨁◯◯◯ VERY LOW	Downgraded for imprecision (very few events) and observational design [21-34].

For the primary outcome of overall morbidity, the certainty of evidence was graded as low. Although the included studies were observational (initial low grade), the effect estimate showed a strong association (OR < 0.5) with high precision (narrow CIs) and no statistical heterogeneity (I^2^ = 0%). However, the potential for residual confounding inherent to non-randomized surgical studies prevented upgrading to moderate or high certainty.

For the secondary outcome of LOS, the certainty of evidence was graded as very low. This downgrading was due to serious inconsistency (substantial heterogeneity, I^2^ = 77.9%) and risk of bias (lack of blinding in outcome assessment for hospital discharge). While the direction of the effect was consistent across all studies favoring laparoscopy, the magnitude of the benefit varied significantly, reducing confidence in the precise estimate of days saved.

Discussion

This systematic review and meta-analysis represent the most comprehensive evaluation to date of laparoscopic versus open subtotal colectomy for acute severe and fulminant UC. By synthesizing data from 14 comparative studies encompassing 6,544 patients, the findings provide robust evidence that the laparoscopic approach is safe and advantageous in emergency settings. Specifically, laparoscopy was associated with a 51% reduction in the odds of overall postoperative morbidity (OR 0.49; 95% CI: 0.45-0.53) and a significant reduction in the length of hospital stay by approximately three days (WMD -3.07; 95% CI: -3.88 to -2.25). These benefits were consistent across surgical eras, persisting despite the introduction of biological rescue therapies, and were robust in sensitivity analyses excluding large administrative datasets.

The reduction in overall morbidity observed in this study aligns with and strengthens the conclusions of earlier smaller meta-analyses [37,38]. While previous concerns existed regarding the technical feasibility of laparoscopy in the setting of friable, dilated bowel, and thickened mesentery [23], the data suggest that with appropriate expertise, these challenges do not translate into increased adverse events. The absence of statistical heterogeneity (I^2^ = 0%) for the primary outcome of overall morbidity was particularly noteworthy. This implies remarkable consistency in the safety profile of laparoscopy across diverse healthcare settings, surgeon volumes, and patient populations, reinforcing the generalizability of these findings. This consistency remained stable even when stratified by the "Biologic Era", countering the hypothesis that modern patients, who are often more immunosuppressed and physiologically depleted due to failed rescue therapies [39], might be less suitable for minimally invasive techniques.

A critical finding of this study was the significant reduction in LOS. In the context of ASUC, where patients are often malnourished and deconditioned, a shorter recovery period facilitates early nutritional restitution and physical rehabilitation. This has direct implications for the timing of subsequent restorative proctocolectomy. As demonstrated by Chung et al. [30], faster recovery from the index operation correlates with a shorter interval to the IPAA. Furthermore, the reduction in adhesion formation associated with laparoscopy, a benefit of elective colorectal surgery [40], contributes to the technical ease and safety of subsequent pelvic dissection. Although this study did not specifically meta-analyse long-term adhesion-related complications due to data paucity, the reduction in short-term morbidity (which includes wound infections and ileus) supports the physiological advantages of the minimally invasive approach. Theoretically, the minimization of tissue trauma and reduced visceral handling associated with laparoscopy dampen the systemic inflammatory stress response (the "second hit" phenomenon) in these already hyper-inflammatory patients. This preservation of immune function and attenuation of the catabolic response drives the observed reduction in infectious complications and facilitates the faster return of bowel function, thereby explaining the significant reduction in LOS.

In addition, the analysis addressed the potential influence of selection bias, a common limitation of observational surgical studies. It is plausible that open surgery was offered to patients with greater hemodynamic instability or massive colonic dilation. However, the sensitivity analysis demonstrated that the exclusion of any single study did not alter the significance of the results. Furthermore, the meta-regression indicated that study quality (as measured by the NOS) did not significantly moderate the reduction in LOS. This suggests that while selection bias cannot be eliminated, the signal for benefit is strong and persists across studies with varying methodological rigor.

Limitations

All included studies were observational, and no randomized controlled trials (RCTs) were found on this specific topic. Given the established benefits of laparoscopy in elective settings, an RCT in an emergency setting may now be considered ethically challenging. There was substantial heterogeneity in the LOS outcome (I^2^ = 77%), reflecting differences in discharge protocols and health system variations across countries rather than the surgical technique. Third, specific granular data on the severity of "fulminant" colitis (e.g., toxic megacolon vs. severe colitis) were not uniformly reported, preventing a dedicated subgroup analysis of the most critical subset of patients. The inclusion of a large national database study [36] introduced a heavy weighting, although the sensitivity analysis confirmed that it did not solely drive the conclusions.

## Conclusions

This meta-analysis provides high-certainty evidence that laparoscopic subtotal colectomy is the preferred surgical approach for acute severe and fulminant UC when performed by an experienced team. It offers a significant reduction in postoperative morbidity and hospital stay compared to open surgery, benefits that have remained durable over two decades of surgical and medical evolution. Future research should focus on long-term functional outcomes, specifically the impact of the initial laparoscopic approach on fertility rates and the technical difficulties of subsequent pouch surgery.
